# Supplementary material for: Molecular forms of Anopheles subpictus and Anopheles sundaicus in the Indian subcontinent
Source: Malar J. 2020 Nov 19;19:417. doi: 10.1186/s12936-020-03492-2 (PMC7678295; doi:10.1186/s12936-020-03492-2)
Supplement: Supplementary file 1 — Additional file 1: Fig. S1. Alignment of rDNA sequences (ITS2 to D3 domain of 28S) of different molecular forms of An. subpictus s.l., and An. sundaicus complex s.l. [file 12936_2020_3492_MOESM1_ESM.pdf]

Additional file 1:

Figure S1. Alignment of rDNA sequences (ITS2 to D3 domain of 28S) of different molecular forms of *An. subpictus* and *An. sundaicus* complex. MW078484: *An. subpictus* Form A (haplotype 1), MW078485: *An. subpictus* Form A (haplotype 2), MW078486: *An. subpictus* Form B, MW078487: *An. subpictus* Form C, MW078488: *An. epiroticus* (variant I) from Myanmar, MW078489: *An. sundaicus* (TK) from Car Nicobar, MW078490: *An. sundaicus* D (variant III). MT068436, MT068425 and MT068434 are representative sequences of *An. subpictus* from Thailand and Indonesia [1]. Dots in alignment represent similarity in nucleotide base with the first row of sequence and dash represents missing nucleotide. The demarcation of ITS2-boundries was done using online ITS2 database tool available at http://its2.bioapps.biozentrum.uni-wuerzburg.de/ (Selig et al., 2008) [2]

```
         --------------------------------------5.8S-------------><--------ITS2-----------
MW078484 CGCATATGGCGCATCGGACGTTTCAACCCGACCGATGCACACATCCTTGAGTGCCTACTAGGTACTGAGAGATTCCTATA [80]
MW078485 ................................................................................
MW078486 ..................................................................TC..TT........
MW078487 ..................................................................TC..TT........
MW078488 ..................................................................TC..TT........
MW078489 ..................................................................TC..TT........
MW078490 ..................................................................TC..TT........
MT068436 ..................................................................TC..TT........
MT068425 ..................................................................TC..TT........
MT068434 ...........Y......................................................TC..TT........

         --------------------------------------ITS2--------------------------------------
MW078484 ACTTGACTACAGACGGGCGCCACAAACGGGCTGACGGGCCATCCGTCGTCCGGCGTGCGACTGTGCAGCATGGCGTGCTC [160]
MW078485 ................................................................................
MW078486 .T.A...................T..T...........TT..........T.............................
MW078487 CT.A...................T..............TT..........T.............................
MW078488 .T.A...................T...............T..........T.............................
MW078489 .T.A...................T...............T..........T.............................
MW078490 .T.A...................T...............T..........T.............................
MT068436 CT.A...................T..............TT..........T.............................
MT068425 .T.A...................T..............TT..........T.............................
MT068434 .T.A...................T..............TT..........T.............................

         --------------------------------------ITS2--------------------------------------
MW078484 GGGTCTCGGCGTGGACCCTTGGGCGCTGAAAGTGGACACT--GTTTG-GCGGCACCTGCGCGTGTGCTCTC---AGTGTT [240]
MW078485 ........................................--.....-.......................---......
MW078486 ....................................T...CT.....A.........TT............CTA.....C
MW078487 ....................................T...CT.....A.........TT............CTA.....C
MW078488 ....................................T...CT.....A.........TT............CTA.....C
MW078489 ....................................T...CT.....A.........TT............CTA.....C
MW078490 ....................................T...CT.....A.........TT............CTA.....C
MT068436 ....................................T...CT.....A.........TT............CTA.....C
MT068425 ....................................T...CT.....A.........TT............CTA.....C
MT068434 ....................................T...CT.....A.........TT............CTA.....C

         --------------------------------------ITS2--------------------------------------
MW078484 GACGTATGGTGAGGGTAGTGTCAAATCGCACGGTTCGACAACA--AGCGTACCGTCGAGTTTGGTGCAATCGGATGCCTA [320]
MW078485 ...........................................--...................................
MW078486 ........................GC........G........CA.......T...........................
MW078487 ........................GC........G........CA.......T...........................
MW078488 ........................GC........G........CA.......T...........................
MW078489 ........................GC........G........CA.......T...........................
MW078490 ........................GC........G........CA.......T...........................
MT068436 ........................GC........G........CA.......T...........................
MT068425 ........................GC........G........CA.......T...........................
MT068434 ........................GC........G........CA.......T...........................

         --------------------------------------ITS2--------------------------------------
MW078484 CTACCATGGGCGGAGCCGGCGTGCATTCAACACTCGACGT------CCTGTATCAACCGGATGCCAACTTGGTTGGTGGT [400]
MW078485 ........................................------..................................
MW078486 .............T..........................GTGCGT......................------------
MW078487 .............T.............A............GCGTGT.............R........------------
MW078488 .............T..........................GCGTGT......................------------
MW078489 .............T..........................GCGTGT......................------------
MW078490 .............T..........................GCGTGT......................------------
MT068436 .............T.............A............GCGTGT......................------------
MT068425 .............T..........................GTGTGT......................------------
MT068434 .............T..........................GTGTGT......................------------
```

```
                ----------------------------------------ITS2----------------------------------------
MW078484 GCCGGCGCAGACAGGACACTGAATCGATCTTGGTGGTACAACCCACATGTGGGTTAGTAGGTAGGTGGTCGATGTGTGCA [480]
MW078485 ..................................................------------..................
MW078486 --------------------------------------------------------------------------------
MW078487 --------------------------------------------------------------------------------
MW078488 --------------------------------------------------------------------------------
MW078489 --------------------------------------------------------------------------------
MW078490 --------------------------------------------------------------------------------
MT068436 --------------------------------------------------------------------------------
MT068425 --------------------------------------------------------------------------------
MT068434 --------------------------------------------------------------------------------

                ----------------------------------------ITS2----------------------------------------
MW078484 GGTGACAACCGGATGCCAGCGATGGCGGTGCCGGCGCACACCAGCACACTCGCCCCTAGGTCGCTTGTTGCGTGTAACGC [560]
MW078485 ................................................................................
MW078486 ----------T.C..--TCA.T...T....T.......G..AG.---..G...G.G..---......AGT..........
MW078487 ----------T.C..--TCA.T...T....T.......G..AG.---..G...G.G..---......AGT..........
MW078488 ----------T.C..TGTCA.T...T....T.......G..AG.---..G...G.G..---......AGT..........
MW078489 ----------T.C..TGTCA.T...T....T.......G..AG.---..G...G.G..---......AGT........K.
MW078490 ----------T.CC.TGTCA.T...T....T.......G..AG.---..G...G.G..---......AGT..........
MT068436 ----------T.C..TGTCA.T...T....T.......G..AG.---..G...G.G..---......AGT..........
MT068425 ----------T.C..--TCA.T...T....T.......G..AG.---..G...G.G..---......AGT..........
MT068434 ----------T.C..--TCA.T...T....T.......G..AG.---..G...G.G..---......AGT..........

                ----------------------------------------ITS2----------------------------------------
MW078484 GTGTGATCCATACACATACCTGTTTGAGCTGTGCGTTGAACACAAGAGGATGAGAGTTGCCAAACA---CACAGACCACA [640]
MW078485 ..................................................................---...........
MW078486 ...C..C........G......C.......A.......------C...TTG...........G..GGAGG.A.T......
MW078487 ...C..C........G......C.......A.......------C...TTG...........G..GGAGG.G.T......
MW078488 ...C..C........G......C...............------C...CTG...........G..GGAG..A.T......
MW078489 ...C..C........G......C...............------C...CTG...........G..GGAG..A.T......
MW078490 ...C..C........G......C...............------C...CTG...........G..GGAG..A.T......
MT068436 ...C..C........G......C.......A.......------C...TTG...........G..GGAGG.G.T......
MT068425 ...C..C........G......C.......A.......------C...TTG...........G..GGAG..A.T......
MT068434 ...C..C........G......C.......A.......------C...TTG...........G..GGAG..A.T......

         ----ITS2---><------------------28S----------------------------------------------
MW078484 ----CTCCAGTAGGCCTCAAGTGATGTGTGACTACCCCCAGAATTTAAGCATATTAATAAGGGGAGGAAGAGAAACCAAC [720]
MW078485 ----............................................................................
MW078486 ACTA............................................................................
MW078487 AACT............................................................................
MW078488 --TA............................................................................
MW078489 --TA............................................................................
MW078490 --TA............................................................................
MT068436 AACT.............................
MT068425 CCTA.............................
MT068434 CCTA.............................

                ----------------------------------------28S-----------------------------------------
MW078484 CGGGATTCCCTGAGTAGCTGCGAGCGAAACGGGAGAAGCCCAGCACGTAGGGACGACGTGGAGACGCGCCTGTCCGGTTC [800]
MW078485 ................................................................................
MW078486 .......................................T...............G.........A..............
MW078487 .......................................T...............G........................
MW078488 .......................................T...............G........................
MW078489 .......................................T...............G........................
MW078490 .......................................T...............G........................

                ----------------------------------------28S-----------------------------------------
MW078484 CGTGTATTGGACCGGTCCGTTATCTATCATGTACAGTGTGCATCAAGTCTAACTTGAAGGTGGCTCATTATCCCATAGAG [880]
MW078485 ................................................................................
MW078486 ...............................C.....CA.........................................
MW078487 ...............................C.....CA.........................................
MW078488 ...............................C.....CA.........................................
MW078489 ...............................C.....CA.........................................
MW078490 ...............................C.....CA.........................................

                ----------------------------------------28S-----------------------------------------
MW078484 GGTGATAGGCCCGTTGCATGCGCGCGCCTGTGCTGGTAGACGGTCGGCTCCAGGGAGTCGTGTTGCTTGATAGTGCAGCA [960]
MW078485 ................................................................................
MW078486 ..............A........A........................................................
MW078487 ..............A........A........................................................
MW078488 ..............A........A........................................................
MW078489 ..............A........A........................................................
MW078490 ..............A........A........................................................
```

```
         ————————————————————————————————————————28S————————————————————————————————————————
MW078484 CTAAGTGGGAGGTAAACTCCTTCTAAGGCTAAATATGACCATGAGACCGATAGCGAACAAGTACCGTGAGGGAAAGTTGA [1040]
MW078485 ................................................................................
MW078486 ..........................A.........CG..........................................
MW078487 ..........................A.........TG..........................................
MW078488 ..........................A.........TG..........................................
MW078489 ..........................A.........TG..........................................
MW078490 ..........................A.........TG..........................................

         ————————————————————————————————————————28S————————————————————————————————————————
MW078484 AAAGCACTCTGAATAGAGAGTCAAATAGTACGTGAAACTGCCTAGGGTACACAAACCCGTTGAACTCAATGATCCGGGCG [1120]
MW078485 ................................................................................
MW078486 ................................................................................
MW078487 ................................................................................
MW078488 ................................................................................
MW078489 ................................................................................
MW078490 ................................................................................

         ————————————————————————————————————————28S————————————————————————————————————————
MW078484 GCGATATTCAGCGGCGGTCGCAAGGCCACCGTGCACTTATCGTTCCGCAGCAAACGGACATCGCGATCCATTACAATAGC [1200]
MW078485 ................................................................................
MW078486 ..............T............G....................................................
MW078487 ..............T............G....................................................
MW078488 ..............T............G....................................................
MW078489 ..............T............G....................................................
MW078490 ..............T............G....................................................

         ————————————————————————————————————————28S————————————————————————————————————————
MW078484 GAGTCGAGCGTTCTGCGCTGCGACCGTCCGGCAT-TACTGGCCCCTGGCTCGTGGTGGACGGCTCCCTAGTAGGGGCGGC [1280]
MW078485 ..................................-.............................................
MW078486 .G....G...C.T.....................G.TA..........................................
MW078487 .GT...G...C.T.....................GCGA..........................................
MW078488 .G....G...C.T.....................GCGA..........................................
MW078489 .G....G...C.T.....................GCGA..........................................
MW078490 .G....G...C.T.....................GCGA..........................................

         ————————————————————————————————————————28S————————————————————————————————————————
MW078484 TTGACGGCCGCACCGAGCGGGGGTCTCCGCGCCTTTCTTCTGAAGGGCGACTGGGTCCGACCGAGCGTGGTGTGCCGCTG [1360]
MW078485 ................................................................................
MW078486 ..TG.................................A..C.......................................
MW078487 ..TG.................................A..C.........M.............................
MW078488 ..TG.................................A..C.......................................
MW078489 ..TG.................................A..C.......................................
MW078490 ..TG.................................A..C.......................................

         ————————————————————————————————————————28S————————————————————————————————————————
MW078484 GAACCGTGATGGATCCGAGCGTGGGGCCGACC--AAGGCTGCCCTC----GTGGCCAGCCAAGCGTGCCCCCAGATCGGC [1440]
MW078485 ................................--............----..............................
MW078486 .........................A....GTTAG....A...T..TGCGAA..TTGT..GTT....T............
MW078487 .........................A....GTTAG....A...T..TGCGAA..TTGT..GTT....T............
MW078488 .........................A....GTTAG....A...T..TGCGAA..TTGT..GTT....T............
MW078489 .........................A....GTTAG....A...T..TGCGAA..TTGT..GTT....T............
MW078490 .........................A....GTTAG....A...T..TGCGAA..TTGT..GTT....T............

         ————————————————————————————————————————28S————————————————————————————————————————
MW078484 GATGAGCAGCATGCATTGAGGCACCTCCGGGACCCGTCTTGAAACACGGACCAAGAAGTCTATCTTGCACGCGAGCCAAT [1520]
MW078485 ................................................................................
MW078486 .....................T..................................................A.......
MW078487 .....................T..................................................A.......
MW078488 .....................T..................................................A.......
MW078489 .....................T..................................................A.......
MW078490 .....................T..................................................A.......

         ————————————————————————————————————————28S————————————————————————————————————————
MW078484 GGGACCGGTGCCACCTTCGGGTGGTGCCGCTCAATCGAACACCCATAGGCGAAGACAACTCGAAGACGCATGTCACGGGA [1600]
MW078485 ................................................................................
MW078486 .......A.A.....GGT.T......T....................................T....TTC.........
MW078487 .......A.A.....GGT.T......T....................................T....TTC.........
MW078488 .......A.A.....GGT.T......T....................................T....TTC.........
MW078489 .......A.A.....GGT.T......T....................................T....TTC.........
MW078490 .......A.A.....GGT.T......T....................................T....TTC.........

         ————————————————————————————————————————28S————————————————————————————————————————
MW078484 TTACGGGTTCGGCATTGGCGCAAGCCTACGTCGGATCCCTCCATCCCAGGGTGCCCCGATACGGGTGGGAGCGGGCCTCC [1680]
MW078485 ................................................................................
MW078486 ...............-...........T....................................................
MW078487 ...............-...........T....................................................
MW078488 ...............-...........T....................................................
MW078489 ...............-...........T....................................................
MW078490 ...............-...........T....................................................
```

```
                              ------------28S------------
MW078484 GGGCGTCGTTCTACCCAGCGGGCATACCCCGAGTGTGCAGGATGCGACCCGAAAGATGGTGAACTATGCCTGATCAGGTT [1760]
MW078485 ................................................................................
MW078486 ................................................................................
MW078487 ................................................................................
MW078488 ................................................................................
MW078489 ................................................................................
MW078490 ................................................................................

                              ------------28S------------
MW078484 GAAGTCAGGGGAAACCCTGATGGAGGACCGAAGCAATTCTGACGTGCAAATCGAT [1815]
MW078485 .......................................................
MW078486 .......................................................
MW078487 .......................................................
MW078488 .......................................................
MW078489 .......................................................
MW078490 .......................................................
```

Reference

1. Wilai P, Ali RSM, Saingamsook J, Saeung A, Junkum A, Walton C, Harbach RE, Somboon P. Integrated systematics of *Anopheles subpictus* (Diptera: Culicidae) in the Oriental Region, with emphasis on forms in Thailand and Sulawesi, Indonesia. Acta Tropica. 2020;208:1-8.

2. Selig C, Wolf M, Müller T, Dandekar T, Schultz J. The ITS2 Database II: homology modelling RNA structure for molecular systematics. Nucleic Acids Res. 2008;36 (Database issue):D377-D380
